# Self-Supervised Spatio-Temporal Network for Classifying Lung Tumor in EBUS Videos

**DOI:** 10.3390/diagnostics15243184

**Published:** 2025-12-13

**Authors:** Ching-Kai Lin, Chin-Wen Chen, Hung-Chih Tu, Hung-Jen Fan, Yun-Chien Cheng

**Affiliations:** 1 Department of Mechanical Engineering, College of Engineering, National Yang Ming Chiao Tung University, Hsin-Chu 300, Taiwan; vanhalen19781205@gmail.com (C.-K.L.);; 2 Department of Medicine, National Taiwan University Cancer Center, Taipei 106, Taiwan; 3 Department of Internal Medicine, National Taiwan University Hospital, Taipei 100, Taiwan; 4 Department of Internal Medicine, National Taiwan University Hsin-Chu Hospital, Hsin-Chu 300, Taiwan; 5 Department of Internal Medicine, National Taiwan University Biomedical Park Hospital, Zhubei 310, Taiwan

**Keywords:** 3D convolutional neural network, endobronchial ultrasound, self-supervised learning, video classification

## Abstract

**Background:** Endobronchial ultrasound-guided transbronchial biopsy (EBUS-TBB) is a valuable technique for diagnosing peripheral pulmonary lesions (PPLs). Although computer-aided diagnostic (CAD) systems have been explored for EBUS interpretation, most rely on manually selected 2D static frames and overlook temporal dynamics that may provide important cues for differentiating benign from malignant lesions. This study aimed to develop an artificial intelligence model that incorporates temporal modeling to analyze EBUS videos and improve lesion classification. **Methods:** We retrospectively collected EBUS videos from patients undergoing EBUS-TBB between November 2019 and January 2022. A dual-path 3D convolutional network (SlowFast) was employed for spatiotemporal feature extraction, and contrastive learning (SwAV) was integrated to enhance model generalizability on clinical data. **Results:** A total of 465 patients with corresponding EBUS videos were included. On the validation set, the SlowFast + SwAV_Frame model achieved an AUC of 0.857, accuracy of 82.26%, sensitivity of 93.18%, specificity of 55.56%, and F1-score of 88.17%, outperforming pulmonologists (accuracy 70.97%, sensitivity 77.27%, specificity 55.56%, F1-score 79.07%). On the test set, the model achieved an AUC of 0.823, accuracy of 76.92%, sensitivity of 84.85%, specificity of 63.16%, and F1-score of 82.35%. The proposed model also demonstrated superior performance compared with conventional 2D architectures. **Conclusions:** This study introduces the first CAD framework for real-time malignancy classification from full-length EBUS videos, which reduces reliance on manual image selection and improves diagnostic efficiency. In addition, given its higher accuracy compared with pulmonologists’ assessments, the framework shows strong potential for clinical applicability.

## 1. Introduction

### 1.1. Background

With the increasing use of low-dose computed tomography (LDCT) for lung cancer screening, peripheral pulmonary lesions (PPLs) have become easier to detect [1]. Accurate diagnosis of PPLs is essential for determining optimal treatment. Endobronchial ultrasound-guided transbronchial biopsy (EBUS-TBB) is a bronchoscopic technique that is widely used for the diagnosis of PPLs, owing to its well-established safety and acceptable risk profile [2,3,4]. During EBUS-TBB, the technique not only confirms the location of PPLs but also provides information about their internal structure [5,6]. Previous studies have attempted to distinguish benign from malignant PPLs based on characteristic EBUS imaging patterns [5,7]. Kurimoto et al. classified EBUS images into three major classes and six subclasses to evaluate the internal structure of lesions and to infer their possible pathological and histological characteristics [5]. Chao et al. later proposed four distinct EBUS image patterns—continuous hyperechoic margin, echogenicity, hyperechoic dots, and concentric circles—to differentiate the nature of PPLs [7]. However, recognizing these sonographic features requires substantial experience, and misinterpretation may reduce diagnostic accuracy and even delay treatment planning. Therefore, an effective approach to presenting EBUS imaging patterns during the procedure is needed.

### 1.2. Literature Review

Computer-aided diagnostic (CAD) systems are computational tools designed to support clinicians in image interpretation and decision-making, thereby enhancing diagnostic accuracy and efficiency. CAD has been applied to ultrasound image analysis in various fields, including breast and thyroid sonography [8,9,10,11]. For EBUS, several CAD systems have also been proposed to assist lesion classification [7,8,12,13]. Chen et al. proposed a CaffeNet-based architecture combined with support vector machines (SVMs) to classify benign and malignant lesions in EBUS images [14]. Their approach achieved an accuracy of 85.4% and an AUC of 0.87, demonstrating the potential of convolutional neural network (CNN) architectures for malignancy assessment in EBUS imaging. Hotta et al. [12] subsequently developed a deep convolutional network model, validated its ability to interpret ultrasound lesion images, and compared its performance with that of multiple experienced physicians. The model achieved an accuracy of 83.3%, outperforming two experts who achieved 73.8% and 66.7%, respectively, thereby confirming that computer-aided interpretation can surpass human performance in this task. However, the datasets used in these studies were manually selected by operators, and all of them employed two-dimensional (2D) architectures.

The 2D structure of CNNs has several limitations that hinder their broader clinical applicability. First, ultrasound images often contain substantial noise, making interpretation difficult. To obtain images suitable for analysis, manual selection and annotation are frequently required, which increases labor demands and reduces practicality. Second, ultrasound imaging during sampling is recorded as real-time video, and lesion characteristics may change dynamically over time. A single 2D image cannot capture all relevant features, and 2D CNNs are unable to establish relationships between features in adjacent frames, leading to the loss of important temporal context.

To overcome these limitations, several studies have investigated the performance of three-dimensional convolutional neural networks (3D CNNs) in ultrasound video recognition tasks, such as echocardiography and fetal ultrasound [15,16]. These studies demonstrated that 3D CNNs outperform 2D models in such applications. The principal advantage of 3D CNNs lies in their ability to incorporate temporal information by processing multiple frames captured at different time points, thereby providing richer contextual information for analysis and decision-making. Implementing a 3D CNN model also has the potential to eliminate the need for physicians to manually select individual frames from ultrasound videos, thereby streamlining the diagnostic process and improving overall efficiency.

### 1.3. Motivation

Based on the aforementioned studies, the integration of CAD systems, particularly those utilizing 3D CNNs, holds great potential to revolutionize the interpretation of EBUS images and overcome the limitations of previous approaches. However, to date, no study has applied a 3D CNN for the interpretation of EBUS images. Therefore, the present study aims to develop a fully automated, video-based CAD system capable of classifying lung lesions directly from raw EBUS recordings, thereby eliminating the need for manual frame selection. The proposed system is designed to assist operators in determining the benign or malignant nature of lung lesions during EBUS-TBB, with the goal of improving diagnostic accuracy and reducing procedural risks. Furthermore, the architecture integrates both temporal and spatial features extracted from the videos and incorporates contrastive learning to mitigate noise interference, aiming to achieve higher classification accuracy compared with existing methods.

To achieve this goal, we address these challenges through three key strategies. First, we employ EBUS video recordings as model inputs to reduce the labor-intensive process of manual frame selection by operators. Second, we design a 3D CNN architecture to capture temporal dynamics within ultrasound sequences, thereby minimizing reliance on individual static images. Third, we aim to mitigate noise interference in predictive outcomes and enhance the robustness and generalizability of model performance across diverse clinical environments.

## 2. Methods

### 2.1. Participants

We retrospectively collected data from patients who underwent EBUS-TBB for the diagnosis of PPLs at the Division of Thoracic Medicine, National Taiwan University Cancer Center, between November 2019 and January 2022. The study protocol was approved by the Institutional Review Board of the National Taiwan University Cancer Center (IRB# 202207064RINB, date 19 August 2022). The requirement for informed consent was waived because only de-identified data were analyzed. All procedures were conducted in accordance with the principles of the Declaration of Helsinki.

Patient characteristics and bronchoscopy findings, including age, gender, and EBUS image patterns, were recorded before or during the procedure. EBUS patterns were classified as type I (homogeneous), type II (hyperechoic dots and linear arcs), and type III (heterogeneous), according to the criteria described by Kurimoto et al. [5].

### 2.2. EBUS-TBB Procedure and Image Collection

All EBUS-TBB procedures were performed by pulmonologists with over 10 years of EBUS experience. After conscious sedation with propofol, midazolam, and fentanyl, a flexible bronchoscope was inserted through a laryngeal mask airway. A 20 MHz radial probe EBUS (UM-S20-17S; Olympus, Tokyo, Japan) was advanced through the working channel into the target bronchus under computed tomography (CT) guidance. Once the target lesion was identified, the probe was moved back and forth slowly for at least 5 s to scan the entire lesion. All EBUS videos were recorded using a medical video recording system (TR2103; TWIN BEANS Co., New Taipei City, Taiwan). During the procedures, two pulmonologists simultaneously assessed the EBUS image patterns.

After confirming the biopsy site, TBB was performed with forceps. Samples were fixed in 10% formalin, paraffin-embedded, and stained with hematoxylin and eosin for evaluation by experienced cytopathologists. Following EBUS-TBB, 25 mL of sterile saline was instilled into the target bronchus, and the retrieved fluid was sent for microbiological analysis. In each EBUS-TBB procedure, only a single target lesion was sampled.

Definitive diagnoses for the EBUS videos were determined based on the final diagnosis of the corresponding PPLs, using cytopathological findings, microbiological results, or clinical follow-up. Histological results that were inconclusive—such as nonspecific fibrosis, chronic inflammation, necrosis, atypical cells, or “suspicious” findings—were classified as non-diagnostic. Benign inflammatory lesions that could not be confirmed pathologically or microbiologically were diagnosed through radiological and clinical follow-up, defined as stability or reduction in lesion size on CT imaging for at least 12 months after EBUS-TBB.

### 2.3. Data Preprocessing

All EBUS videos were sampled at a rate of one frame every 0.4 s. Every eight consecutive frames were stacked into a single 3D image, hereafter referred to as a clip, which served as the model input. To maintain temporal continuity and avoid splitting adjacent frames into different clips, a 50% overlap was applied between neighboring clips. As a result, each clip encompassed 3.2 s of video information, with a 1.6 s interval between successive clips.

The images were cropped to 960 × 960 pixels and subsequently downsampled to 224 × 224 pixels to reduce computational demands. To mitigate overfitting, horizontal flipping was applied with a probability of 50% as a data augmentation strategy. In addition, the Noise CutMix method was employed in selected experiments during SwAV model training to assess its impact on noise robustness.

### 2.4. Noise CutMix

**Figure 1:**
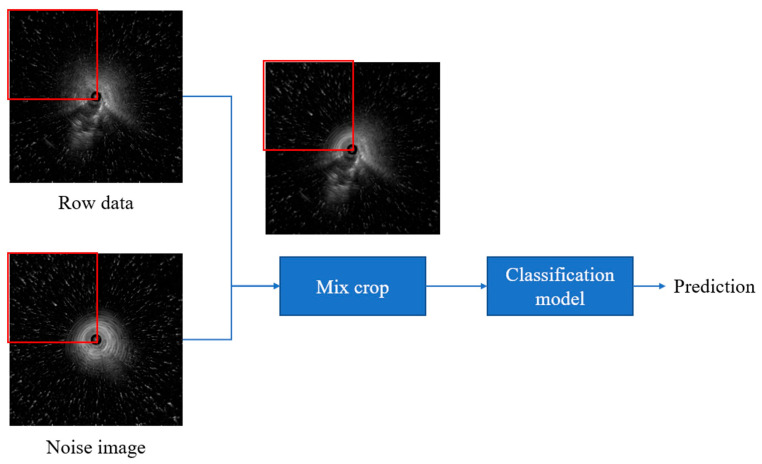
Noise CutMix process.

In this study, we introduced “Noise CutMix”, a variation of CutMix that replaces image regions with noise patches by adding additional noise to the image, the model’s ability to extract features from images containing noise is enhanced (Figure 1). We first randomly capture 200 images from segments containing no lesions only air echoes. Then, to simulate real-world fan-shaped noise, we segment the radial ultrasound images in 90-degree increments, forming four rectangles: upper left, upper right, lower left, and lower right. Next, we randomly replace the relative positions in the original images to create images with noise. This method is used for training SwAV contrastive learning and subsequent validation of noisy data to ensure that the images contain noise from air echoes. Because our goal was to simulate the types of noise encountered during actual surgery, fan-shaped noise was generated, so no other data augmentation strategies were used.

### 2.5. SlowFast

In video recognition, 3D networks better capture temporal dynamics than 2D models but incur higher computational costs. To balance efficiency and performance, Feichtenhofer et al. [15] proposed the SlowFast dual-stream architecture, where the Slow pathway (3D ResNet50 backbone) emphasizes static spatial features with fewer frames and more channels, while the Fast pathway captures temporal dynamics with more frames and fewer channels. In our implementation, the Fast pathway samples at four times the frequency of the Slow pathway with 32 input frames, and its feature maps are fused with the Slow pathway via 3D convolution to integrate spatial and temporal information.

### 2.6. SwAV

Contrastive learning is an unsupervised approach that improves clustering by pulling similar (positive) samples closer in feature space while pushing dissimilar (negative) samples apart. Unlike conventional methods, SwAV eliminates the need for negative samples [16]. It compares differently augmented views of the same image by assigning them to prototype cluster centers and swapping their soft-label codes for prediction. The SwAV method first calculates the similarity between the feature and K cluster centers (prototypes) to generate codes belonging to soft labels. Then, these codes are used as pseudo-labels to exchange and predict each other. The loss calculation of this method is shown in Equations (1)–(3).

(1) L(z_t_, z_s_) = l(z_t_, q_s_) + l(z_s_, q_t_),

(2) l(z_t_, q_s_) = −∑_k_ q_s_^(k)^ logP_t_^(k)^,

(3) P_t_^(k)^ = {exp(1/τ × z_t_^T^ × c_k_)}/{∑_(k^′)_ exp(1/τ × z_t_^T^ × c_(k^′)_)},

### 2.7. Model Overview

**Figure 2:**
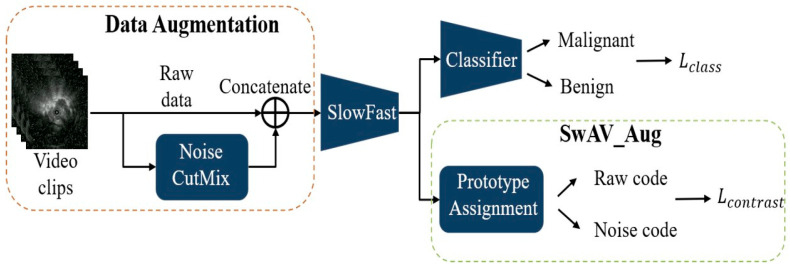
SlowFast + SwAV_Aug.

**Figure 3:**
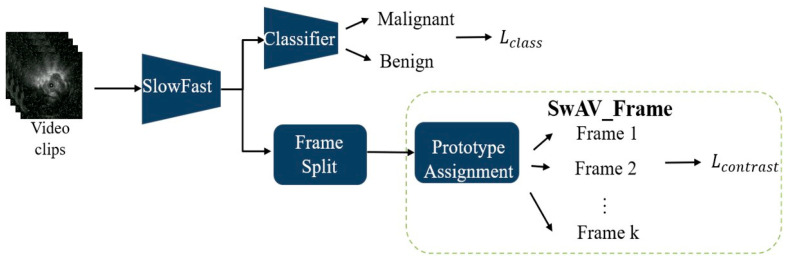
SlowFast + SwAV_Frame.

During training, the model integrates two modules: SlowFast and the SwAV. SwAV is applied in two ways: SwAV_Aug (Figure 2), which indicates that the positive pairs in the SwAV module are generated through data augmentation. The classification loss is computed by comparing the predicted scores of benign and malignant lesions—produced by the classifier—with the corresponding pathological ground truth labels. The contrastive loss, on the other hand, is derived from the proposed SwAV_Aug approach. In this method, data augmentation is performed using Noise CutMix during input processing. Each augmented and original image is passed through the SlowFast network to extract their respective feature representations. The original and noise-augmented images serve as positive pairs, whose feature embeddings are compared in the clustering space to compute the contrastive loss. This mechanism aims to enhance the feature consistency of images originating from the same source and improve the model’s robustness to noise; and SwAV_Frame (Figure 3), which the positive samples are derived from frames within the same clip. Since these frames are temporally continuous, we expect them to be predicted as belonging to the same class, thereby preventing transient noise from disrupting classification consistency. Therefore, these frames are treated as mutual positive pairs for computing the contrastive loss, encouraging the model to maintain consistent predictions across temporally adjacent frames. SlowFast is trained jointly with SwAV, optimizing both classification and contrastive losses.

### 2.8. Statistical Analysis

We evaluated temporal information and model generalizability by comparing 2D and 3D neural networks for classifying benign versus malignant lung lesions from EBUS videos. The effects of contrastive learning and attention mechanisms were also assessed under different training strategies. To account for variable video lengths, segment-level predictions were averaged to generate case-level results, with malignancy assigned when the probability exceeded 0.5. Performance was measured by AUC, accuracy, sensitivity, specificity, and F1-score.

The F1-score, defined as the harmonic mean of precision and recall, was calculated as:

(4) F1-score = 2 × (Precision × Recall)/(Precision + Recall),

Precision is calculated as:

(5) Precision = (True Positive)/(True Positive + False Positive),

Recall (also referred to as sensitivity) is defined as:

(6) Recall = (True Positive)/(True Positive + False Negative),

Pulmonologists’ assessments on the validation set served as the benchmark, with type II and type III EBUS image patterns classified as malignant, and type I patterns classified as benign. We utilized SPSS version 22.0 (IBM, SPSS, Chicago, IL, USA) for statistical analysis.

## 3. Results

### 3.1. Patients and EBUS Videos

A total of 465 patients with 465 corresponding PPL-related EBUS videos were included in the dataset, which was subsequently divided into two categories. Of these, 309 videos collected between November 2019 and April 2021 were randomly split at an 8:2 ratio into 247 training cases (175 malignant and 72 benign) and 62 validation cases (44 malignant and 18 benign). The test set consisted of 156 cases obtained between April 2021 and January 2022, including 100 malignant and 56 benign cases. The details of patient and video data are summarized in Table 1 and Table S1.

### 3.2. Model Performance on the Validation Set

For 2D CNN baselines, training was performed at the frame level because these architectures do not incorporate temporal modeling. Each EBUS video clip was decomposed into individual frames, which were independently processed by the 2D network. The clip-level output was obtained by averaging the predicted probabilities across all frames belonging to the same clip. During training, the loss for each clip was computed from the averaged frame-level prediction to ensure consistency between the learning objective and the clip-level evaluation used in the 3D models. This strategy allows fair comparison between 2D and 3D architectures while preserving the frame-based nature of 2D CNNs.

As shown in Table 2, moving from 2D CNNs to 3D architectures improves the classification of radial EBUS-TBB videos, reflecting the importance of temporal information in ultrasound imaging. The SlowFast architecture further enhances performance by leveraging both high-resolution spatial cues and dense temporal dynamics.

Introducing contrastive learning produces distinct effects depending on how positive pairs are formed. Augmentation-based pairing tends to increase sensitivity but leads to more false positives, and recall increases while specificity decreases, indicating a bias toward predicting malignancy. In contrast, frame-based pairing promotes a more balanced prediction pattern by improving specificity while maintaining high sensitivity and decreasing recall, suggesting that temporal consistency encourages a more conservative decision boundary.

Both contrastive-learning variants outperform conventional 2D and baseline 3D models and show higher overall consistency than pulmonologists. These findings emphasize two key points: temporal modeling is essential for EBUS video analysis, and contrastive learning systematically shifts decision behavior—augmentation-based methods lean toward malignancy, whereas frame-based methods encourage benign-oriented predictions. This provides practical flexibility for adjusting sensitivity–specificity trade-offs according to clinical needs.

### 3.3. Model Performance on the Test Set

As shown in Table 3, most evaluation metrics (e.g., AUC and F1 score) of all models showed a decrease. However, although the model using SwAV_Aug exhibited extremely poor specificity, it maintained the best performance, while the model using SwAV_Frame maintained more than 50% specificity, showing no significant decline in performance and demonstrating some generalization ability. Despite the decline in other metrics, these results, compared to the performance of pulmonologists, indicate that these models have potential clinical application value.

## 4. Discussion

This retrospective study demonstrated that our proposed system, which analyzes EBUS videos, is highly effective in distinguishing between benign and malignant processes, outperforming conventional 2D models and even surpassing pulmonologists’ assessments.

Previous studies have shown that convolutional neural networks (CNNs) can achieve high efficiency in classifying EBUS-TBB lesions. Chen et al. combined a CaffeNet-based architecture with support vector machines (SVMs) to distinguish benign from malignant lesions, achieving an accuracy of 85.4% and an AUC of 0.87 [14]. Hotta et al. [12] developed a deep convolutional network that demonstrated superior accuracy, sensitivity, specificity, positive predictive value, and negative predictive value compared with bronchoscopists. The model achieved an accuracy of 83.3%, outperforming two experienced physicians (73.8% and 66.7%) [12]. Consistent with these findings, our proposed system also exceeded pulmonologists’ diagnostic accuracy, sensitivity, and F1-score. These results suggest that CAD systems may have stronger interpretative capability for EBUS images than even experienced bronchoscopists and could be particularly helpful in evaluating lesions that are more challenging to interpret during clinical EBUS examinations.

However, previous studies on EBUS lesion classification have predominantly relied on 2D CNN architectures applied to static ultrasound images [12,13,14]. These approaches were based on manually selected frames and lacked temporal modeling, which limits their applicability in real-time clinical workflows. As shown in Table 2, our results confirm these limitations of 2D approaches. Although certain 2D CNN models, such as VGG19 and ViT, achieved high sensitivity (≥97%), their specificity was markedly lower (≤33%), indicating a bias toward predicting malignancy.

To our knowledge, this study presents the first end-to-end CAD architecture specifically developed for real-time classification of full-length EBUS videos in clinical settings. By incorporating temporal information, particularly through the motion-sensitive SlowFast network, our 3D models achieved a more balanced diagnostic performance between sensitivity and specificity. The SlowFast model demonstrated the highest AUC (0.864) among all evaluated architectures, with a sensitivity of 95.45% and an F1-score of 87.50%, outperforming 2D models and even surpassing pulmonologists’ assessments in overall diagnostic accuracy. These results highlight the clinical value of video-based analysis, in which temporal cues such as probe motion, tissue deformation, and vascular pulsation provide essential contextual information for accurate lesion characterization.

Human visual processing studies have shown that even complex natural-scene categorization can occur within approximately 150 ms [17], which is 6.7 FPS, suggesting that near-real-time feedback is essential for practical integration into bronchoscopy workflows. The inference speed of our model (15–30 FPS) falls within this latency range according to our setup, indicating that the proposed system is computationally feasible for real-time use.

The results in Table 2 illustrate how different contrastive learning strategies affect model bias and robustness. Incorporating SwAV_Aug maximized sensitivity (100%) but substantially lowered specificity (22.22%), reflecting a bias toward malignancy predictions. Such a strategy may be advantageous in high-sensitivity screening scenarios where minimizing false negatives is critical. In contrast, SwAV_Frame achieved the highest specificity (55.56%) and the best F1-score (88.17%) among the SlowFast-based models, providing a more balanced trade-off that is better suited for confirmatory diagnostic applications.

Table 3 shows that generalizability testing revealed performance declines across all models—particularly in specificity—when applied to external datasets. Notably, the SwAV_Frame configuration maintained specificity comparable to that of pulmonologists’ assessments under domain shift, demonstrating greater resistance to overfitting and improved reliability in real-world clinical settings. Compared with pulmonologists, whose validation set performance yielded an F1-score of 79.07% and specificity of 55.56%, several AI configurations—including SlowFast and SwAV_Frame—not only exceeded pulmonologists’ sensitivity (≥93.18% vs. 77.27%) but also achieved comparable specificity in select cases. These findings highlight the potential of AI-assisted diagnosis to enhance sensitivity without compromising specificity in routine bronchoscopy practice.

Although the 95% confidence intervals of AUC values could not be obtained due to the lack of raw prediction scores, the relative ranking of model performance remains valid, as all models were evaluated on the same dataset using identical procedures. The conclusions based on accuracy, sensitivity, specificity, and F1-score—each of which has well-defined confidence intervals—therefore remain statistically reliable.

The integration of temporal modeling and contrastive learning offers a versatile diagnostic framework adaptable to different clinical needs. High-sensitivity configurations—such as those incorporating SwAV_Aug—may be particularly valuable for early detection or screening of high-risk patients, where minimizing missed malignancies is critical. Conversely, high-specificity models—such as those based on SwAV_Frame—could be applied in confirmatory settings to reduce unnecessary biopsies and their associated risks.

Importantly, the model’s diagnostic performance was comparable to—and in some configurations exceeded—that of experienced pulmonologists, particularly with respect to sensitivity. Unlike 2D approaches that require manual frame selection, our system processes full-length EBUS videos without interrupting the procedure. These features highlight the potential of the system as a real-time decision-support tool to enhance diagnostic consistency, reduce inter-operator variability, and guide optimal biopsy site selection during bronchoscopy.

Our study has several limitations. First, this study was based on a single-center dataset with retrospectively collected data. It is well recognized that evaluating a model on a single real-world dataset may not fully capture its performance across diverse healthcare settings, and testing on multiple datasets would help validate its generalizability. In addition, the relatively limited histopathologic heterogeneity and the uneven distribution of histopathologic subtypes among the training, validation, and testing sets may also have influenced the accuracy of the model. However, this study is the first to utilize EBUS video recordings for model training, and currently, no publicly available dataset exists for this purpose. Operator variability may also introduce potential bias, particularly when operators have differing levels of experience with EBUS procedures. Moreover, although the proposed models demonstrate potential for real-time deployment, their integration into routine bronchoscopy workflows has not yet been validated. Future multi-center, prospective studies are needed to confirm robustness across diverse patient populations, imaging platforms, and operator techniques, as well as to evaluate usability, and clinical impact.

Second, we did not use a surgical gold standard to define all final pathologies; however, all benign processes without definitive cytopathologic or microbiologic evidence were clinically followed for at least 12 months and characterized using chest CT. This approach is more consistent with real-world clinical practice. Finally, although AI-assisted diagnosis showed promise in matching or exceeding clinical performance, its optimal role—whether as a screening tool, confirmatory aid, or both—remains to be clarified. Integration into clinical workflows, together with operator training and performance monitoring, will be essential to ensure sustained benefits.

## 5. Conclusions

Our study developed a comprehensive model integrating the SlowFast architecture with the SwAV contrastive learning approach to classify lung lesions as benign or malignant from EBUS videos. The findings demonstrate that incorporating temporal information enhances model performance, while self-supervised contrastive learning markedly improves both accuracy and generalizability. Using the SlowFast + SwAV_Frame architecture, the model achieved high diagnostic efficiency on both the validation and test sets, surpassing pulmonologists’ assessments across multiple metrics. We believe that our proposed artificial intelligence-assisted diagnostic system, which operates without interrupting the procedure, has the potential to support pulmonologists during EBUS-TBB by shortening the time required to differentiate benign from malignant lesions and by identifying optimal biopsy sites. This may reduce biopsy failure and the number of sampling attempts, lower procedure-related risks, and ultimately improve the diagnostic yield of EBUS-TBB.

In addition to achieving strong diagnostic performance, the incorporation of self-supervised contrastive learning substantially enhances model generalizability while reducing reliance on large manually annotated datasets. This property is particularly advantageous in ultrasound-based applications, where labeling is time-consuming and operator-dependent. These strengths further support the potential clinical applicability and scalability of the proposed framework.

To our knowledge, this is also the first study to present an end-to-end CAD architecture specifically developed for the real-time classification of full-length EBUS videos in clinical settings. However, this study was based on a single-center dataset, and its retrospective design may limit the generalizability of the findings. A multicenter, prospective study is needed to confirm the model’s robustness across diverse patient populations.

## Figures and Tables

**Table 1:** Baseline characteristics of patients and their corresponding EBUS videos.

Characteristics	Total	Training Set	Validation Set	Test Set
**Patients**				
Number	465	247	62	156
Age (years old, range)	65.1 (20–92)	65.6 (21–92)	65.2 (33–88)	64.2 (20–90)
Gender (male, %)	227 (48.8)	119 (48.2)	27 (43.5)	81 (51.9)
Indication (initial diagnosis, %)	357 (76.8)	189 (76.5)	50 (80.6)	118 (75.6)
**EBUS video (%)**				
Image pattern				
Type I	174 (37.4)	89 (36.0)	25 (40.3)	60 (38.5)
Type II	33 (7.1)	16 (6.5)	2 (3.2)	15 (9.6)
Type III	258 (55.5)	142 (57.5)	35 (56.5)	81 (51.9)
Malignancy	319 (68.6)	175 (70.9)	44 (71.0)	100 (64.1)
Lung adenocarcinoma	211 (45.4)	119 (48.2)	29 (46.8)	63 (40.3)
Lung squamous cell carcinoma	31 (6.7)	15 (6.1)	9 (14.5)	7 (4.4)
Small cell lung cancer	14 (3.0)	8 (3.2)	0 (0)	6 (3.8)
Other non-small cell lung cancer	32 (6.9)	15 (6.1)	2 (3.2)	15 (9.6)
Non-lung cancer malignancy	31 (6.7)	18 (7.3)	4 (6.5)	9 (5.8)
Benign process	146 (31.4)	72 (29.1)	18 (29.0)	56 (35.9)
TB/NTM infection	20 (4.3)	11 (4.5)	1 (1.6)	8 (5.1)
Fungal infection	8 (1.7)	3 (1.2)	1 (1.6)	4 (2.6)
Bacterial infection	16 (3.4)	9 (3.6)	1 (1.6)	6 (3.8)
Interstitial lung disease	40 (8.6)	21 (8.5)	8 (12.9)	11 (7.1)
Hamartoma	1 (0.2)	1 (0.4)	0 (0)	0 (0)
Benign inflammation	61 (13.1)	27 (10.9)	7 (11.3)	27 (17.3)

EBUS, endobronchial ultrasound; NTM, nontuberculous mycobacteria; TB, tuberculosis.

**Table 2:** Classification performance of 2D models, 3D models, and pulmonologists’ assessments on the validation set.

Model	AUC *	Accuracy (%)	Sensitivity (%)	Specificity (%)	F1-Score (%)
**2D models**					
GoogLeNet	0.807	70.97	100.00	0.00	83.02
2D ResNet50	0.837	72.58	72.73	5.26	83.81
VGG19	0.850	77.42	97.97	27.78	83.33
ViT	0.831	79.03	97.73	33.33	86.87
**3D models**					
3D ResNet50	0.850	75.81	88.64	44.44	83.87
SlowFast	0.864	80.65	95.45	44.44	87.50
**Our models**					
SlowFast + SwAV_Aug	**0.872**	77.42	**100.00**	22.22	86.27
SlowFast + SwAV_Frame	0.857	**82.26**	93.18	**55.56**	**88.17**
Pulmonologists’ assessments	-	70.97	77.27	55.56	79.07

* AUC, area under the curve.

**Table 3:** Classification performance of 2D models, 3D models, and pulmonologists’ assessments on the test set.

Model	AUC *	Accuracy (%)	Sensitivity (%)	Specificity (%)	F1-Score (%)
**2D models**					
GoogLeNet	0.717	65.38	97.98	8.77	78.23
2D ResNet50	0.765	64.74	97.98	7.02	77.91
VGG19	0.780	69.87	89.90	35.09	79.11
ViT	0.781	67.95	93.94	22.81	78.81
**3D models**					
3D ResNet50	0.804	73.72	91.92	42.11	81.61
SlowFast	0.812	67.31	92.93	22.81	78.30
**Our models**					
SlowFast + SwAV_Aug	**0.828**	**86.38**	**97.98**	8.77	78.23
SlowFast + SwAV_Frame	0.823	76.92	84.85	**63.16**	**82.35**
Pulmonologists’ assessments	-	67.95	73	58.93	74.49

* AUC, area under the curve.

## Data Availability

The datasets presented in this article are not readily available due to IRB restrictions. In accordance with the requirements of National Taiwan University Cancer Center, all data analyses were conducted within the hospital’s designated Intelligent Research Center. The data cannot be transferred or publicly released outside of this secure research environment. Requests to access the datasets should be directed to the corresponding author, and will be considered following institutional regulations and IRB approval.

## Supplementary Materials

The following supporting information can be downloaded at: https://www.mdpi.com/article/10.3390/diagnostics15243184/s1, Table S1: Detailed histopathologic subtypes of other non-small cell lung cancers

## Abbreviations

The following abbreviations are used in this manuscript:

2D	Two-Dimensional
3D	Three-Dimensional
AUC	Area Under the Curve
CAD	Computer-Aided Diagnostic system
CNN	Convolutional Neural Network
CT	Computed Tomography
EBUS	Endobronchial Ultrasound
NTM	Nontuberculous Mycobacteria
PPLs	Peripheral Pulmonary Lesions
SVM	Support Vector Machine
TB	Tuberculosis
TBB	Transbronchial Biopsy
ViT	Vision Transformer
