# Supplementary material for: Self-Supervised Spatio-Temporal Network for Classifying Lung Tumor in EBUS Videos
Source: Diagnostics (Basel). 2025 Dec 13;15(24):3184. doi: 10.3390/diagnostics15243184 (PMC12731602; doi:10.3390/diagnostics15243184)
Supplement: Supplementary file 1 [file diagnostics-15-03184-s001.zip › diagnostics-3971819-supplementary.pdf]

Table S1 Detailed histopathologic subtypes of other non–small cell lung cancers.

Characteristics	Total	Training set	Validation set	Test set
Other non-small cell lung cancer (N)	32	15	2	15
Poorly differentiated carcinoma (%)	11 (34.4)	8 (53.3)	0 (0)	3 (20)
Pleomorphic carcinoma (%)	7 (21.9)	3 (20)	1 (50)	3 (20)
Adenosquamous carcinoma (%)	2 (6.3)	0 (0)	0 (0)	2 (13.3)
Large cell carcinoma (%)	1 (3.1)	0 (0)	0 (0)	1 (6.7)
Mucoepidermoid carcinoma (%)	4 (12.5)	1 (6.7)	0 (0)	3 (20)
Neuroendocrine tumor (%)	2 (6.3)	0 (0)	0 (0)	2 (13.3)
Adenoid cystic carcinoma (%)	5 (15.6)	3 (20)	1 (50)	1 (6.7)

N = number.
